# Causes of nephrotic syndrome in Sweden: The relevance of clinical presentation and demographics

**DOI:** 10.3389/fneph.2023.1026864

**Published:** 2023-03-17

**Authors:** Anneli Jönsson, Thomas Hellmark, Mårten Segelmark, Anna Forsberg, Karl Dreja

**Affiliations:** ^1^ Department of Clinical Sciences, Lund University, Lund, Sweden; ^2^ Department of Health Science, Lund University, Lund, Sweden; ^3^ Department of Thoracic Surgery, Skåne University Hospital, Lund, Sweden; ^4^ Department of Clinical Sciences, Lund University, Skane University Hospital, Malmö, Sweden

**Keywords:** nephrotic syndrome, epidemiology, albuminuria, glomerulonephritis, diabetic nephropathy, membranous nephropathy

## Abstract

**Background:**

Many pathological processes can disrupt the integrity of the glomerular capillary wall and cause a massive leakage of protein, resulting in nephrotic syndrome (NS). Clinical parameters such as age, sex, renal function, presence of diabetes, and how NS is defined influence the spectrum of underlying diseases. In this study, we examine how these parameters interact.

**Methods:**

Age, sex, hematuria, proteinuria, plasma creatinine plasma albumin levels, and final diagnosis were retrieved for all adult patients with NS as an indication for biopsy and/or massive albuminuria in conjunction with low plasma albumin from the biopsy module of the Swedish Renal Registry (SRR) between 2014 and 2019. A basic calculator was developed to demonstrate the importance of clinical presentation in relation to the likelihood of having a specific diagnosis.

**Results:**

A total of 913 unique patients were included in the study. Diabetic nephropathy (DN) and membranous nephropathy (MN) (both found in 17% of patients) were the most common diagnoses. With a stringent definition of NS, MN and minimal change nephropathy (MCN) increased in proportion. Among the cohort as a whole, MCN was the most frequent diagnosis in women and those < 50 years of age (found in 21% and 17%, respectively). In the case of patients aged between 50 and 70 years, those with chronic kidney disease stage 4, and those with negative dipstick tests for hematuria, the most common underlying disease was DN (in 23%, 30%, and 21% of cases, respectively). Among those with high-grade hematuria (dipstick grade 3 or 4), membranoproliferative glomerulonephritis was the most common diagnosis (14%), closely followed by IgA nephropathy (13%). Focal segmental glomerulosclerosis (9.7%) was less common than in many comparable studies.

**Conclusion:**

Clinical parameters have a profound impact on the likelihood of different diagnoses in adult patients with NS. Differences in clinical practice and study inclusion criteria may be more important than genetic background and environmental factors when explaining differences between studies in different parts of the world.

## Introduction

Massive leakage of proteins in the kidneys associated with edema is referred to as nephrotic syndrome (NS) (1). The incidence of NS is three or four cases per 100,000 per year (2–4), and NS is in many reports the most frequent indication for renal biopsy (5). In a recent study (6) on patients’ perspectives on suffering from NS, we found that NS is perceived as a highly complex condition. Many patients describe the illness experience as being a stranger in an unfamiliar world of symptoms and medical treatments. This perception is at least partly shared by physicians, as the heterogeneity of the condition is a problem. Understanding how different clinical presentations are associated with different diagnoses and pathological processes is probably helpful.

The distribution of causes of NS varies between countries and over time, which complicates comparisons of the influence of clinical features on the distribution of underlying diseases. Membranous nephropathy (MN) is the most common cause of NS in India (7), Spain (8), and Denmark (9). Focal segmental glomerulosclerosis (FSGS) is the most common cause in the USA (10), whereas IgA nephropathy (IgAN) is the most common cause in Czechia (11) and mesangial proliferative glomerulonephritis (MPGN) is the most common cause in Lebanon (12).

There are existing studies based on national registers (4, 9, 11, 13), regional registries (8, 14), and data from single centers (7, 10). Most studies have not focused specifically on NS, and some have included only scarce clinical data. Some studies have excluded secondary causes of NS, such as diabetic nephropathy (DN) and systemic lupus erythematosus (SLE) (15, 16). There have been few attempts to analyze how demographic factors and clinical presentation affect the likelihood of different diagnoses among adult patients with NS. The aim of this study is to describe the causes of NS in the Swedish population and analyze how the spectrum is dependent on factors that are readily available before the results of a biopsy. To this end, we retrieved data on biopsy indication, albuminuria, hematuria, chronic kidney disease (CKD) stage, age, and sex from the renal biopsy module of the Swedish Renal Registry (SRR).

## Materials and methods

### Data source

Data were retrieved from the biopsy module of the SRR. The organization of the SRR is described in a previous publication (17); the biopsy module was launched in 2015 for the prospective registration of biopsies from native kidneys (18). It is possible to enter the details of older biopsies retrospectively, and this has been done in a systematic fashion at some hospitals for 2013 and 2014. For biopsy indication, one of the following five alternatives must be chosen: NS, acute nephritic syndrome, other acute kidney injury, CKD stage 1 or 2, and CKD stages 3–5. CKD stage 1 or 2 is essentially what in some other studies is referred to as urine abnormalities. The Swedish term that translates as “acute nephritic syndrome” encompasses subacute disease that elsewhere is classified as rapidly progressive glomerulonephritis. If a patient fulfills the criteria for more than one biopsy indication, the responsible clinician must choose the most compelling indication for the procedure. The result of the biopsy is entered both with a SNOMED (Systematized Nomenclature of Medicine) code from the pathologist and with an ERA-EDTA (European Renal Association—European Dialysis and Transplant Association) code for primary renal disease, as chosen by the nephrologist when combining clinical features, laboratory results, and biopsy results. Multiple diagnoses can be assigned, but the one best explaining the biopsy indication should be entered as the main diagnosis.

### Study population

We included data from biopsies performed in adults (≥ 18 years) between 1 January 2014 and 31 December 2019. Three searches were performed in the biopsy module of the SRR: (i) biopsy indication NS; (ii) urine albumin-to-creatinine ratio > 300 mg/mmol; and (iii) urine albumin > 3.5 g/24 hours. Data retrieved from the registry at the time of biopsy included patient age, sex, weight, renal diagnosis, blood pressure, dipstick hematuria reading, plasma creatinine level, estimated glomerular filtration rate (eGFR; calculated using the CKD-EPI equation), plasma C-reactive protein (CRP), plasma albumin, urine albumin-to-creatinine ratio, urine albumin (24-hour collection), and serum hemoglobin. Patients with a biopsy indication other than NS were excluded from the study if plasma albumin was > 30 g/L. Data on the ongoing pharmacological treatment of hypertension are included in the database but were not retrieved for this study. The registry does capture data on acute complications of renal biopsy but not on late complications, such as infections and thromboembolic events.

We grouped the ERA-EDTA codes version 2018 for primary renal diseases (19) into 12 groups (**Table 1**). A total of 46 patients (19 women and 27 men) had had more than one biopsy. In these cases, clinical data and diagnosis were taken from the last-performed biopsy. The reason for performing a repeat biopsy is most often that the initial biopsy yielded insufficient material and was unable to provide a clear diagnosis.

**Table 1:** Grouping of renal disease and ERA-EDTA diagnosis codes.

Renal disease	ERA-EDTA codes
Focal segmental glomerulosclerosis (FSGS)	1061, 1267, 1308, 1320, 1354
IgA nephropathy (IgAN)	1128, 1144, 1515
Membranoproliferative glomerulonephritis (MPGN)	1222, 1233, 1246
Membranous nephropathy (MN)	1185, 1192, 1205, 1214
Minimal change nephropathy (MCN)	1100
Other glomerulonephritis	1251, 1331, 1349, 1365, 1377
Diabetic nephropathy (DN)	2328, 2337, 2344
Systemic lupus erythematosus (SLE)	1493
Plasma cells dyscrasias (PCD)	2521, 2584, 2597, 2606
Vascular disease	2359, 2363, 2385, 2411, 2448
Vasculitis	1383, 1401, 1417, 1429, 1472
Other renal diagnosis	1570, 1591, 1897, 1930, 2014, 2257, 2288, 2509, 2513, 2623, 2634, 2668, 2681, 2760, 3380, 3398, 3419, 3442, 3564

### Diagnostic calculator

A diagnostic calculator was developed based on the frequencies of each of the categorical clinical parameters for each diagnosis (**Supplementary Material**). Age, sex, level of hematuria, CKD stage, and known diabetes were used as parameters, as they are well established and known to differ between diagnoses. For example, the distribution of FSGS in the cohort according to age was as follows: 47% of patients were aged 18–49 years, 27% were aged 50–70 years, and 26% were over 70 years. In addition, 49% of patients were male and 51% female. Similar calculations were made for degree of hematuria, known diabetes, and CKD stage. When entering the five parameters, one can calculate the relative chance of each diagnosis by multiplying the frequency for each of the five parameters, and thereafter dividing this by the sum of the products for all diagnoses. The ratio between the calculated chance and the chance of having the specific diagnosis in the entire cohort generated the relative difference. All relative differences below zero were inverted and multiplied by –1 in order to graphically present positive and negative fold changes on a single representative scale. The parameters were equally weighted, and no multinomial logistics were applied.

### Statistical analysis

All analysis was performed using IBM SPSS Statistics (version 26.0; IBM Corporation, Armonk, NY, USA) software for Mac. Categorical variables (sex, grade of hematuria, etc.) were expressed as frequencies and percentages. Chi-squared or Fisher’s exact tests were used, when appropriate, to compare group differences between categorical variables. The parametric continuous variable (age) was expressed as mean ± standard deviation (SD). An independent-samples *t*-test was used to compare groups. Non-parametric continuous variables (laboratory parameters, weight, and blood pressure) were expressed as medians and interquartile ranges. A Mann–Whitney *U*-test was used to compare the two independent groups (NS and other indications). *p*-values < 0.05 were considered statistically significant.

## Results

### Study population

**Figure 1:**
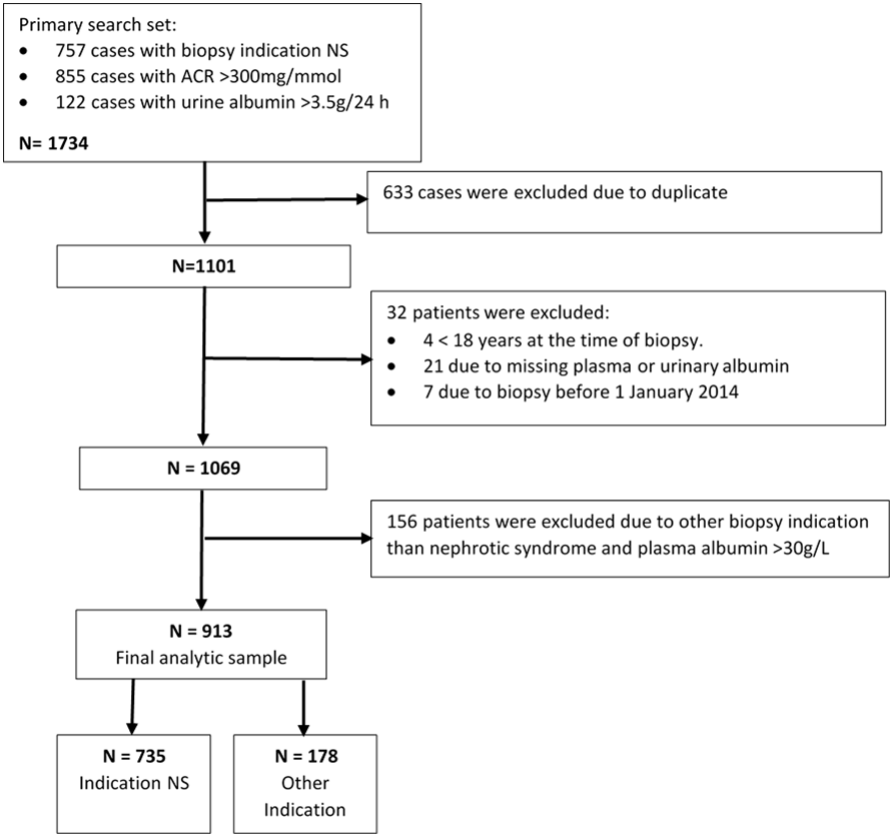
Flow chart of study population.

Our primary search criteria generated a total of 1,734 biopsy entries. After the removal of duplicates, repeat biopsies, incomplete data sets, and patients not fulfilling the inclusion criteria, 913 unique patients were included in the analyses (shown in **Figure 1**). There were 735 patients with a biopsy indication of NS. Within the NS group, there were 487 (66%) patients who, on the day of biopsy, fulfilled strict laboratory criteria for NS [i.e., urine albumin-to-creatine ratio (ACR) > 300 g/mol and plasma albumin < 30 g/L], and 248 (34%) who did not. There were 178 patients who had laboratory parameters compatible with NS but in whom the main biopsy indication was not NS. In this “other indication” group, the most common indication for biopsy was CKD stage 3–5 (48.3%), followed by acute nephritic syndrome (30.9%), CKD stage 1 or 2 (11.2%), and other acute kidney injury (9.6%).

### Indication NS vs. other indication

There were only small and insignificant differences in age and sex distributions between those with a biopsy indication of NS and those with another indication. There were, however, substantial differences in plasma creatinine concentration, which in the “other indication” group was more than twice that in the NS group; the opposite was the case for eGFR. The indication NS group also had lower systolic blood pressure (median 135 vs. 140 mmHg) and higher levels of blood hemoglobin (median 126 vs. 113 g/L) and included a lower proportion of patients with hematuria of grade 3 or 4 (26.2% vs. 43.5%) (**Table 2**).

**Table 2:** Demographic data and clinical characteristics at the time of biopsy.

Variables	All patients (*n *= 913)	Indication NS (*n *= 735)	Other indication (*n *= 178)	*p*-value
Age (years), mean ± SD	56.3 ± 17.56	56.3 ± 17.74	56.7 ± 16.83	0.250 ^a^
Sex, % (*n*)				
Male	57.8 (528)	57.1 (420)	60.7 (108)	0.221^b^
Female	42.2 (385)	42.9 (315)	39.3 (70)	
Plasma creatinine (µmol/L)	122 (80–210)	106 (77–179)	233 (139–460)	< 0.001^c^
eGFR (mL/min/1.73 m^2^)	48 (25–75)	56 (31–80)	24 (11–41)	< 0.001^c^
Plasma albumin (g/L)	24 (18–28)	23 (17–28)	25 (21–28)	0.065 ^c^
Urine albumin-to-creatinine ratio (mg/mmol)	486 (331–728)	477 (310–724)	530 (374–742)	0.021^c^
Blood hemoglobin (g/L)	123 (107–138)	126 (109–141)	113 (100–130)	< 0.001^c^
Weight (kg)	80 (68–95)	79 (68–94)	84 (69–98)	0.068^c^
Systolic blood pressure (mmHg)	135 (123–149)	135 (121–147)	140 (130–150)	0.001^c^
Diastolic blood pressure (mmHg)	79 (70–85)	78 70–85	80 (71–85)	0.125^c^
Grade of hematuria, % (*n*)				
0	22.1 (188)	23.3 (159)	17.1 (29)	< 0.001 ^b^
1 or 2	48.2 (411)	50.4 (344)	39.4 (67)	
3 or 4	29.7 (253)	26.2 (179)	43.5 (74)	

Values are presented as medians (interquartile ranges) unless otherwise specified.

a Comparison with independent sample t-test on indication NS—other indication.

b Differences in categorical variables were tested using chi-squared tests.

c Mann–Whitney U-test.

Overall, the three most common diagnoses were DN (17.5%), MN (17.2%), and minimal change nephropathy (MCN) (15.3%). The biggest difference between the NS and the “other indication” group was seen for MCN (18.2% vs. 2.2%). Major differences in the same direction were noted also for MN, FSGS, and SLE. Differences in the opposite direction were seen for IgAN, vasculitis, and vascular diseases (**Table 3**).

**Table 3:** Causes of NS depending on indication for biopsy.

Diagnosis	Overall, % (*n*)	Indication NS, % (*n*)	Other indication, % (*n*)
	(*n *= 913)	(*n *= 735)	(*n *= 178)
FSGS	8.4 (77)	9.7 (71)	3.4 (6)
IgAN	6.7 (61)	4.9 (36)	14.0 (25)
MPGN	5.7 (52)	5.4 (40)	6.7 (12)
MN	17.2 (157)	19.0 (140)	9.6 (17)
MCN	15.3 (140)	18.5 (136)	2.2 (4)
Other glomerulonephritis	3.0 (27)	2.7 (20)	3.9 (7)
DN	17.5 (160)	16.5 (121)	21.9 (39)
SLE	3.9 (36)	4.5 (33)	1.7 (3)
PCD	6.6 (60)	7.1 (52)	4.5 (8)
Vascular diseases	5.7 (52)	4.4 (32)	11.2 (20)
Vasculitis	3.1 (28)	1.8 (13)	8.4 (15)
Other renal diagnosis	6.7 (61)	5.3 (39)	12.4 (22)
No diagnosis	0.2 (2)	0.3 (2)	0.0 (0)

Next, we compared the patients in the NS group who had laboratory features compatible with NS (*n* = 487) with those not fulfilling the proteinuria (i.e., urine albumin-to-creatinine ratio > 300 mg/mmol or urine albumin > 3.5 g/24 hours) and/or albumin (plasma albumin ≤ 30 g/L) criteria set up in this study at the time of biopsy (*n* = 248). There were only minor differences between the groups in clinical parameters such as age, sex, renal function, and hematuria (data not shown). The relative proportions of each diagnosis among patients not fulfilling laboratory criteria tended to be similar to those in the “other indication” group, that is, there were fewer cases of MN and MCN and more cases of IgAN and vasculitis. However, in contrast to the “other indication” group, there tended to be more cases of SLE and FSGS (data not shown).

### Demographics

The two diagnoses whose frequency increased most with age were vascular disease (from 1.3% among those aged 18–49 years to 10.0% among those aged above 70 years) and plasma cell dyscrasias (PCDs) (ranging in frequency from 1.0% among those aged 18–49 years to 13.4% among those aged above 70 years). Diseases whose frequency decreased with age included IgAN, MCN, and SLE. DN showed the highest proportion in the middle age group (i.e., those aged 50–70 years), at 22.8%, being lower among both the youngest and oldest age groups. The diagnosis with the most stable proportion in the different age groups was MPGN (**Table 4**).

**Table 4:** Causes of NS in different subgroups based on age, sex, grade of hematuria, and CKD.

Diagnosis	Age group, % (*n*)			Sex, % (*n*)		Grade of hematuria, % (*n*)			Stage of CKD, % (*n*)			
	18–49 years (*n *= 304)	50–70 years (*n *= 378)	> 70 years (*n *= 231)	Male (*n *= 528)	Female (*n *= 385)	0 (*n *= 188)	1 or 2 (*n *= 411)	3 or 4 (*n *= 253)	1 or 2 (*n *= 362)	3 (*n *= 265)	4 (*n *= 188)	5 (*n *= 98)
FSGS	11.8 (36)	6.9 (26)	6.5 (15)	8.3 (44)	8.6 (33)	11.7 (22)	8.8 (36)	6.7(17)	10.8 (39)	9.4 (25)	6.4 (12)	1.0 (1)
IgAN	12.2 (37)	4.5 (17)	3.0 (7)	7.0 (37)	6.2 (24)	2.1 (4)	4.6 (19)	13.4 (34)	5.8 (21)	6.8 (18)	8.5 (16)	6.1 (6)
MPGN	5.3 (16)	5.8 (22)	6.1 (14)	4.5 (24)	7.3 (28)	2.1 (4)	1.9 (8)	14.2 (36)	2.8 (10)	6.4 (17)	10.1 (19)	6.1 (6)
MN	13.8 (42)	18.5 (70)	19.5 (45)	19.5 (103)	14.0 (54)	11.2 (21)	23.4 (96)	13.4 (34)	26.0 (94)	15.5 (41)	10.6 (20)	2.0 (2)
MCN	20.7 (63)	13.8 (52)	10.8 (25)	13.8 (73)	17.4 (67)	22.3 (42)	15.3 (63)	9.1 (23)	27.1 (98)	9.1 (24)	7.4 (14)	4.1 (4)
Other GN	3.0 (9)	2.6 (10)	3.5 (8)	2.3 (12)	3.9 (15)	2.7 (5)	1.2 (5)	5.1 (13)	2.2 (8)	3.4 (9)	1.1 (2)	8.2 (8)
DN	13.2 (40)	22.8 (86)	14.7 (34)	20.5 (108)	13.5 (52)	21.3 (40)	20.7 (85)	5.9 (15)	6.6 (24)	21.5 (57)	29.8 (56)	23.5 (23)
SLE	9.2 (28)	1.1 (4)	1.7 (4)	0.8 (4)	8.3 (32)	3.2 (6)	3.4 (14)	5.9 (15)	6.1 (22)	4.2 (11)	0.5 (1)	2.0 (2)
PCD	1.0 (3)	6.9 (26)	13.4 (31)	6.6 (35)	6.5 (25)	6.9 (13)	8.0 (33)	4.7 (12)	6.6 (24)	8.7 (23)	5.3 (10)	3.1 (3)
Vascular diseases	1.3 (4)	6.6 (25)	10.0 (23)	6.6 (35)	4.4 (17)	10.1 (19)	6.3 (26)	2.4 (6)	1.7 (6)	6.4 (17)	10.1 (19)	10.2 (10)
Vasculitis	1.0 (3)	4.0 (15)	4.3 (10)	3.0 (16)	3.1 (12)	0.0 (0)	0.5 (2)	9.5 (24)	0.3 (1)	2.6 (7)	2.1 (4)	16.3 (16)
Other renal diagnosis	7.6 (23)	6.3 (24)	6.1 (14)	6.8 (36)	6.5 (25)	6.4 (12)	5.6 (23)	9.5 (24)	3.9 (14)	5.7 (15)	8.0 (15)	17.3 (17)
No diagnosis	0.0 (0)	0.3 (1)	0.4 (1)	0.1 (1)	0.3 (1)	0.0 (0)	0.2 (1)	0.0 (0)	0.3 (1)	0.4 (1)	0.0 (0)	0.0 (0)

Data are presented as percentages (numbers) in columns for each subgroup *CKD (chronic kidney disease). CKD stages 1 and 2 = eGFR ≥ 60 mL/min/1.73 m^2^; CKD stage 3 = eGFR 30–59 mL/min/1.73 m^2^; CKD stage 4 = eGFR 15–29 mL/min/1.73 m^2^; CKD stage 5 = eGFR< 15 mL/min/1.73 m^2^.

Overall, men outnumbered women 1.5 to 1, in relative terms. The diseases showing the largest differences in frequency and which were more common in men were DN (20.5% vs. 13.5%) and MN (19.5% vs. 14.0%). Diagnoses that were more common in women included SLE (8.3% vs. 0.8%), MPGN (7.3% vs. 4.5%), and MCN (17.4% vs. 13.8%) (**Table 4**).

### Hematuria and CKD stage

When categorizing patients on the basis of the level of dipstick hematuria (grade 0, grade 1 or 2, and grade 3 or 4), diseases whose frequency increased in proportion with increasing hematuria were vasculitis (0%, 0.5%, and 9.5%, respectively), MPGN (2.1%, 1.9%, and 14.2%, respectively), and IgAN (2.1%, 4.6%, and 13.4%, respectively). Diseases whose frequency decreased as hematuria increased were DN (21.3%, 20.7%, and 5.9%, respectively) and MCN (22.3%, 15.3%, and 9.1%, respectively). In the case of MN, the frequency of disease was relatively highest among those with moderate levels of hematuria (23.4%, compared with 11.2% among those with negative dipstick tests and 13.4% among those with grade 3 or 4 hematuria) (**Table 4**).

When comparing the relative proportions among those determined, upon biopsy, to have CKD stage 1 or 2 and those with CKD stage 5, the largest decreases were seen for MN (from 26.0% to 2.0%) and MCN (from 27.1% to 4.1%). The largest increases were seen for vasculitis (from 0.3% to 16.3%) and other vascular diseases (from 1.7% to 10.2%); DN exhibited an increase from 6.6% in CKD stages 1 and 2 to 29.8% in CKD stage 4 but fell back to 23.5% in CKD stage 5. A relatively stable proportion in different CKD stages was seen for IgAN, being highest in CKD stage 4, at 8.5%, and lowest in CKD stages 1 and 2, at 5.8% (**Table 4**).

### Nephrosis diagnosis calculator

A diagnostic calculator was developed based on the frequencies of clinical parameters for each diagnosis. Age, sex, level of hematuria, known diabetes, and CKD stage were used as parameters. When these are entered, the calculator provides the likelihood of different diagnoses and how the percentage changes compared with no knowledge of clinical data (**Supplementary Material**).

**Figure 2:**
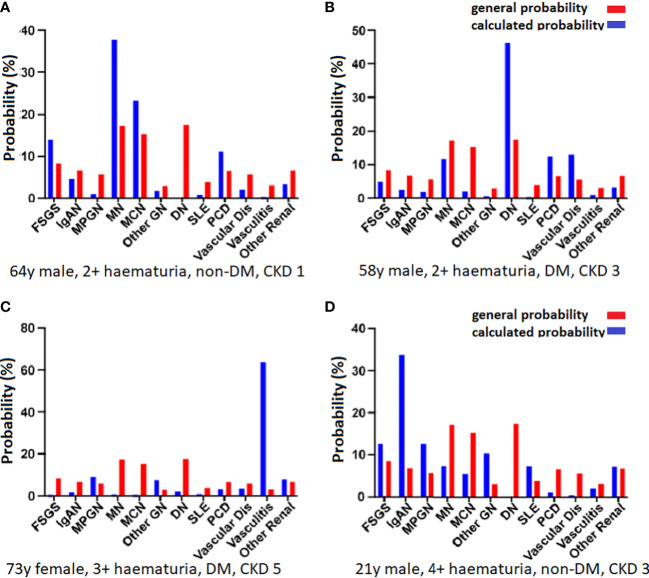
Calculated probabilities for four patients. The red bars indicate the overall probability without entering any specific patient data and the blue bars show the calculated probabilities. The parameters were age, sex, diabetes status, hematuria, and CKD stage. **(A)** In a 64-year-old man with 2+ hematuria, no known diabetes, and CKD stage 1, the most likely diagnosis is MN (38%), followed by MCN (23%) and FSGS (14%). **(B)** In a 58-year-old man with 2+ hematuria, known diabetes, and CKD stage 3, the most likely diagnosis is DN (47%), followed by vascular disease (13%), PCD (13%), and MN (12%). **(C)** In a 73-year-old woman with 3+ hematuria, known diabetes, and CKD stage 5, there is a very high probability of vasculitis (64%), and a lower risk of MPGN (9%), and other renal disease (8%). **(D)** A 21-year-old man with 4+ hematuria, no known diabetes, and CKD stage 3 is most likely to have a diagnosis of IgAN (34%), followed by MPGN and FSGS (13% each).

For example, in the case of a 30-year-old woman with 3+ hematuria, CKD stage 2, and no known diabetes, SLE is the most likely diagnosis (54% chance), followed by IgAN (12%) and MCN (9%). However, if the patient is male, the most probable diagnosis is IgAN (28%), followed by MCN (16%) and FSGS (14%). Four other examples are shown in **Figure 2**. To evaluate the calculator, all patients with complete data were entered into the calculator. The biopsy-verified diagnosis was found among the top three diagnoses in 66% of cases, ranging from 19% for the collective diagnosis group “Other renal diagnoses” to 89% for DN.

## Discussion

In this study, we present data on the causes of NS in Sweden and how this identification of cause is affected by clinical presentation, basic clinical features, and demographic factors. These data can be used to facilitate the identification of the most probable diagnosis in cases when biopsy cannot be performed because of contraindications. In the cohort with the indication NS, the most frequent diagnoses were MN (19%) and MCN (18.5%), followed by DN (16.5%). However, the proportion varied considerably when subsets based on certain clinical parameters were studied.

### Main findings and comparison with previous studies

Definitions of disease and inclusion criteria are always crucial in studies concerning disease incidence. In patients with high levels of albuminuria and low levels of serum albumin, and in whom the clinician-determined indication for biopsy was not NS, DN and IgAN were the most frequent diagnoses. This is in line with the findings of other studies (4, 7–9, 20–23). In contrast, a study from Lebanon (12) found that MPGN was the most common diagnosis in NS, accounting for 27% of cases.

There were differences in disease patterns on regional and national levels owing to socioeconomic factors and ethnicity (10). Compared with some other studies (7, 10, 12, 15, 16), we found a high proportion of patients with DN and a low proportion with FSGS. It is well known that FSGS is associated with both obesity (24) and African ancestry (10, 16). The SRR database contains no information on ethnicity, but in the general Swedish population, approximately 82% are of European descent. Among the 18% of non-European ancestry, the largest population comes from the Middle East and relatively few from western Africa, which might explain some of these differences.

Clinical practice, for instance for differing indications for biopsy, will also affect the reported patterns. DN was found to be a common cause of NS (16.5%) and was the most common diagnosis in patients with high levels of albuminuria and “other indication” for biopsy (21.9%). We believe that this high share is explained by differences in clinical practice and inclusion criteria for studies. Bandi et al. (7) described a wide spectrum of kidney diseases in a South Indian population. A small number of patients with CKD or acute kidney injury were diagnosed with DN, and only 1.4% of patients with NS had DN as the main diagnosis. This may reflect a different preference when it comes to renal biopsy; in contrast to other diagnoses, DN does not require biopsy for diagnosis. A difference in clinical practice is also suggested by the large difference in the mean age of the patients, being 23.3 (7) and 56.3 years in our study, respectively. The cause of NS also varies with patient age. Among younger patients (i.e., those aged < 50 years). MCD, IgAN, and SLE were relatively common. In older patients (i.e., those aged > 50 years), rates of MN, DN, and PCD increased. This result agrees with the findings of most other studies (4, 20–22). PCD and MN were more frequent in patients older than 80 years than in other age groups (data not shown).

Data limitations and differing inclusion criteria should also be considered when comparing studies. Haas et al. (15) and Korbet et al. (16) excluded secondary causes such as diabetes mellitus, SLE, and vasculitis. O’Shaughnessy et al. (10) studied an American population and selected biopsies with glomerular disease diagnoses, divided into nephritic and nephrotic subtypes, not considering clinical parameters, and not including less common causes of NS (e.g., IgAN, vasculitis, lupus nephritis, tubulointerstitial nephritis, hypertensive nephropathy, Alport syndrome, and pre-eclampsia). Approximately 25% of patients in our study would have been excluded using the same criteria.

### Hematuria

Hematuria was common in all groups and diagnoses. Among patients with NS as indication for biopsy, only 22% had no hematuria and 30% had high-grade hematuria (grade 3 or 4). In the latter group, MPGN, IgAN, MN, and vasculitis were the four most frequent diagnoses. Hematuria is thus not a discriminator for NS, and there appears to be an overlap between nephritic and nephrotic syndromes. For example, IgAN, a common cause of nephritic syndrome, was the seventh most common diagnosis in patients with NS as an indication for biopsy.

### Diagnosis calculator

To demonstrate the ability of clinical data to predict the diagnosis in individual patients, we developed a basic tool. By calculating the probability of the different diagnoses based on four clinical parameters (age, sex, hematuria, known diabetes, and CKD stage), we can show the probabilities based on the distribution within these subgroups. As shown in **Figure 2**, these probabilities exhibit substantial differences. Nonetheless, it is important to keep in mind that the generated probabilities should not be used to set a diagnosis. Adding data from other cohorts would make it possible to enter more parameters, such as serology, proteinuria, or previous disease history, and refine the results. More data are also needed to make predictions of more rare diagnoses. The calculator may be further developed by using multinominal logistic regression or machine learning to calculate different weightings of the entered parameters and could potentially be used to find the diagnosis in cases when it is not possible to perform a biopsy.

### Limitations and strengths

This study has several limitations. The cohort stems from a single European country with a primarily European population, and we do not have data on ethnicity. Indication for biopsy is entered at the discretion of individual clinicians and has only been validated to a limited degree. Within the indication NS group, almost one-third did not fulfill stringent criteria for NS at the time of biopsy. The reason for this includes day-to-day fluctuations in the results of laboratory tests and the effect of treatments such as angiotensin-converting enzyme (ACE) inhibition and steroids. If a patient at any time point had NS, the indication for biopsy remained unchanged, even if the syndrome had been partly resolved before the procedure was performed. There is an ongoing validation process in the SRR: it has been shown that the indications assigned are correct in > 95% of cases (unpublished data). However, we cannot exclude mistakes in the registration procedures.

The study also has important merits. It contains a large number of cases collected over a relatively short period of time, and thus reflects the country’s current situation. We provide data both on patients with a defined set of laboratory values and patients with a clinical diagnosis of NS and compare the differences between these two inclusion criteria. We thereby provide the entire spectrum of NS.

## Conclusion

We present a thorough examination of data from a national registry of biopsy data in which we found that clinical parameters have a profound impact on the likelihood of different diagnoses in adult patients with NS. Differences in clinical practice and inclusion criteria in studies may overshadow genetic background and environmental exposers when comparing data from different parts of the world.

## Data availability statement

The raw data supporting the conclusions of this article will be made available by the authors, without undue reservation.

## Ethics statement

The studies involving human participants were reviewed and approved by the regional ethics approval board in Stockholm, Sweden (reference number: 2018/1591–31/2). The patients/participants provided their written informed consent to participate in this study.

## Author contributions

AJ, KD, TH, and MS set up the retrieval strategy, retrieved data from the SRR, and analyzed the data. All authors contributed to the design of the study and wrote the manuscript. All authors contributed to the article and approved the submitted version.

## References

[1] Orth SR Ritz E. The nephrotic syndrome. N Engl J Med (1998) 338:1202–11. doi: 10.1056/NEJM199804233381707 9554862

[2] Hull RP Goldsmith DJ. Nephrotic syndrome in adults. BMJ (2008) 336:1185–9. doi: 10.1136/bmj.39576.709711.80 PMC2394708 18497417

[3] Jegatheesan D Nath K Reyaldeen R Sivasuthan G John GT Francis L. Epidemiology of biopsy-proven glomerulonephritis in Queensland adults. Nephrol (Carlton) (2016) 21:28–34. doi: 10.1111/nep.12559 26154936

[4] Rivera F López-Gómez JM Pérez-García R. Clinicopathologic correlations of renal pathology in Spain. Kidney Int (2004) 66:898–904. doi: 10.1111/j.1523-1755.2004.00833.x 15327378

[5] Fiorentino M Bolignano D Tesar V Pisano A Van Biesen W D'Arrigo G. Renal biopsy in 2015–from epidemiology to evidence-based indications. Am J Nephrol (2016) 43:1–19. doi: 10.1159/000444026 26844777

[6] Jönsson A Hellmark T Forsberg A. Persons' experiences of suffering from nephrotic syndrome. J Ren Care (2020) 46:45–51. doi: 10.1111/jorc.12307 31746128 PMC7328708

[7] Bandi VK Nalamati A Kasinaboina B Chundru SS. Epidemiologic data of biopsy-proven renal diseases: Experience from a single center in south India. Saudi J Kidney Dis Transpl (2019) 30:478–91. doi: 10.4103/1319-2442.256855 31031384

[8] Verde E Quiroga B Rivera F López-Gómez JM. Renal biopsy in very elderly patients: Data from the Spanish registry of glomerulonephritis. Am J Nephrol (2012) 35:230–7. doi: 10.1159/000336307 22343659

[9] Heaf J Løkkegaard H Larsen S. The epidemiology and prognosis of glomerulonephritis in Denmark 1985-1997. Nephrol Dial Transplant (1999) 14:1889–97. doi: 10.1093/ndt/14.8.1889 10462267

[10] O'Shaughnessy MM Hogan SL Poulton CJ Falk RJ Singh HK Nickeleit V. Temporal and demographic trends in glomerular disease epidemiology in the southeastern united states, 1986-2015. Clin J Am Soc Nephrol (2017) 12:614–23. doi: 10.2215/CJN.10871016 PMC5383393 28325866

[11] Rychlík I Jancová E Tesar V Kolsky A Lácha J Stejskal J. The Czech registry of renal biopsies. occurrence of renal diseases in the years 1994-2000. Nephrol Dial Transplant (2004) 19:3040–9. doi: 10.1093/ndt/gfh521 15507479

[12] Karnib HH Gharavi AG Aftimos G Mahfoud Z Saad R Gemayel E. A 5-year survey of biopsy proven kidney diseases in Lebanon: Significant variation in prevalence of primary glomerular diseases by age, population structure and consanguinity. Nephrol Dial Transplant (2010) 25:3962–9. doi: 10.1093/ndt/gfq302 PMC3108367 20525974

[13] Schena FP. Survey of the Italian registry of renal biopsies. Frequency of the renal diseases for 7 consecutive years. The Italian group of renal immunopathology. Nephrol Dial Transplant (1997) 12:418–26. doi: 10.1093/ndt/12.3.418 9075118

[14] Zaza G Bernich P Lupo A. Incidence of primary glomerulonephritis in a large north-Eastern Italian area: A 13-year renal biopsy study. Nephrol Dial Transplant (2013) 28:367–72. doi: 10.1093/ndt/gfs437 23223218

[15] Haas M Meehan SM Karrison TG Spargo BH. Changing etiologies of unexplained adult nephrotic syndrome: A comparison of renal biopsy findings from 1976-1979 and 1995-1997. Am J Kidney Dis (1997) 30:621–31. doi: 10.1016/s0272-6386(97)90485-6 9370176

[16] Korbet SM Genchi RM Borok RZ Schwartz MM. The racial prevalence of glomerular lesions in nephrotic adults. Am J Kidney Dis (1996) 27:647–51. doi: 10.1016/s0272-6386(96)90098-0 8629623

[17] Welander G Sigvant B. Validating vascular access data in the Swedish renal registry SRR. J Vasc Access (2021) 22:629–34. doi: 10.1177/1129729820954737 32951502

[18] Peters B Nasic S Segelmark M. Clinical parameters predicting complications in native kidney biopsies. Clin Kidney J (2020) 13:654–9. doi: 10.1093/ckj/sfz132 PMC7467621 32905412

[19] Venkat-Raman G Tomson CR Gao Y Cornet R Stengel B Gronhagen-Riska C. New primary renal diagnosis codes for the ERA-EDTA. Nephrol Dial Transplant (2012) 27:4414–9. doi: 10.1093/ndt/gfs461 PMC3520087 23175621

[20] Nie P Chen R Luo M Dong C Chen L Liu J. Clinical and pathological analysis of 4910 patients who received renal biopsies at a single center in northeast China. BioMed Res Int (2019). doi: 10.1155/2019/6869179 PMC6457280 31032355

[21] Zhou FD Shen HY Chen M Liu G Zou WZ Zhao MH. The renal histopathological spectrum of patients with nephrotic syndrome: An analysis of 1523 patients in a single Chinese centre. Nephrol Dial Transplant (2011) 26:3993–7. doi: 10.1093/ndt/gfr166 21515637

[22] Hu R Quan S Wang Y Zhou Y Zhang Y Liu L. Spectrum of biopsy proven renal diseases in central China: A 10-year retrospective study based on 34,630 cases. Sci Rep (2020) 10:10994. doi: 10.1038/s41598-020-67910-w 32620914 PMC7335090

[23] Sugiyama H Yokoyama H Sato H Saito T Kohda Y Nishi S. Japan Renal biopsy registry: The first nationwide, web-based, and prospective registry system of renal biopsies in Japan. Clin Exp Nephrol (2011) 15:493–503. doi: 10.1007/s10157-011-0430-4 21437579

[24] Kambham N Markowitz GS Valeri AM Lin J D'Agati VD. Obesity-related glomerulopathy: An emerging epidemic. Kidney Int (2001) 59:1498–509. doi: 10.1046/j.1523-1755.2001.0590041498.x 11260414

